# The subcellular localization of neuronal nitric oxide synthase determines the downstream effects of NO on myocardial function

**DOI:** 10.1093/cvr/cvx002

**Published:** 2017-02-01

**Authors:** Ricardo Carnicer, Silvia Suffredini, Xing Liu, Svetlana Reilly, Jillian N. Simon, Nicoletta C. Surdo, Yin H. Zhang, Craig A. Lygate, Keith M. Channon, Barbara Casadei

**Affiliations:** Division of Cardiovascular Medicine, Radcliffe Department of Medicine, University of Oxford, L6 West Wing, John Radcliffe Hospital, Headley Way, Headington, Oxford. OX3 9DU, UK

**Keywords:** Neuronal NOS, Nitric oxide, Cardiomyocyte, Calcium channel, Excitation–contraction coupling

## Abstract

**Aims:**

In healthy hearts, the neuronal nitric oxide synthase (nNOS) is predominantly localized to the sarcoplasmic reticulum (SR), where it regulates the ryanodine receptor Ca^2+ ^release channel (RyR2) and phospholamban (PLB) phosphorylation, and to a lesser extent to the sarcolemmal membrane where it inhibits the L-type Ca^2+ ^current (*I*_Ca_). However, in failing hearts, impaired relaxation and depressed inotropy are associated with a larger proportion of nNOS being localized to the sarcolemmal membrane. Whether there is a causal relationship between altered myocardial function and subcellular localization of nNOS remains to be assessed.

**Methods and results:**

Adenoviruses (AdV) encoding for a human nNOS.eGFP fusion protein or eGFP were injected into the left ventricle (LV) of nNOS^−/−^ mice. nNOS.eGFP localized to the sarcolemmal and t-tubular membrane and immunoprecipitated with syntrophin and caveolin-3 but not with RyR2. Myocardial transduction of nNOS.eGFP resulted in a significantly increased NOS activity (10-fold, *P* < 0.01), a 20% increase in myocardial tetrahydrobiopterin (BH4) (*P* < 0.05), and a 30% reduction in superoxide production (*P* < 0.001). LV myocytes transduced with nNOS.eGFP showed a significantly lower basal and β-adrenergic stimulated *I*_Ca_, [Ca^2+^]_i_ transient amplitude and cell shortening (vs. eGFP). All differences between groups were abolished after NOS inhibition. In contrast, nNOS.eGFP had no effect on RyR nitrosylation, PLB phosphorylation or the rate of myocardial relaxation and [Ca^2+^]_i_ decay.

**Conclusion:**

Our findings indicate that nNOS-mediated regulation of myocardial excitation–contraction (E–C) coupling is exquisitely dependent on nNOS subcellular localization and suggests a partially adaptive role for sarcolemmal nNOS in the human failing myocardium.

## 1. Introduction

A ‘neuronal’ isoform of nitric oxide synthase (nNOS or NOS1) in the mammalian myocardium has been reported to regulate cardiac inotropy and relaxation by decreasing *I*_Ca_,^1^^,^^2^ increasing ryanodine receptor Ca^2+ ^release channel (RyR2) open probability^3^^,^^4^ and promoting PLB phosphorylation.^5^^,^^6^ Furthermore, nNOS-derived NO has been shown to inhibit myocardial superoxide production,^4^^,^^7–9^ preserve left ventricle (LV) diastolic function^4^^,^^10^ and protect against adverse LV remodelling and arrhythmias after myocardial infarction.^2^^,^^11^^,^^12^ In healthy hearts, nNOS is predominantly located to the sarcoplasmic reticulum (SR),^13^ where it co-localizes with the RyR2,^14^ and to a lesser extent to the sarcolemmal membrane.^15–17^ However, in the presence of heart failure or ischemia/reperfusion injury, myocardial nNOS is upregulated and preferentially located to the sarcolemmal membrane where it binds to caveolin-3 (Cav-3).^18–21^ It has been suggested that rearrangement in nNOS subcellular localization in the presence of heart failure may play an important role in the downstream effects of NO on cardiac function; if that were the case, the sarcolemmal localization of nNOS in failing hearts would be expected to result in a reduction in PLB phosphorylation, contributing to impaired relaxation and depressed contractility secondary to a reduction in Ca^2+ ^influx *via I*_Ca_. However, the diffusibility of NO and the close proximity of the ion channels and transporters involved in excitation–contraction (E–C) coupling may mitigate against the importance of nNOS subcellular localization. To address these questions, we investigated the effect of adenovirus (AdV)-mediated sarcolemmal-specific expression of nNOS on basal and β-adrenergic inotropy, Ca^2+ ^handling, and myocardial superoxide production in LV myocytes from nNOS^−^^/^^−^ mice.

## 2. Methods

NOS1^−^^/^^−^ mice (3–4-months old) of both sexes were used in these experiments.^22^ In some protocols their wild type (WT) littermates were used as controls. All experiments were in accordance with the UK Home Office Guidance on the Operation of Animals (Scientific Procedures) Act, 1986 and approved by the local Ethics Committee.

### 2.1 Identification of nNOS isoforms in the LV myocardium

Total RNA was extracted from skeletal muscle and the LV using the RNeasy Qiagen kit and cDNA generated by reverse transcriptase reaction. One set of primers (Forward 5′- gagaatggggagaaattcgg - 3′ and Reverse 5′- ctgagaacctcacattggcc - 3′) was designed to identify the μ and α nNOS isoforms.^22^^,^^23^ Amplicons of 196 and 298 bp were obtained for the μ and α isoform, respectively.

### 2.2 *In vivo* AdV-mediated gene transfer

Replication-deficient AdV encoding for a human α-nNOS-eGFP fusion protein, α-nNOS, or eGFP only, under the control of the CMV promoter, were propagated in 293 cells and purified by double CsCl gradient ultracentrifugation. Viral titre was determined by plaque assay. Thoracotomy was performed in isofluorane (2%) anaesthetized nNOS^−^^/^^−^ mice and the AdV (10^8^ PFU) was delivered via multiple LV intramyocardial microinjections (six injections of 10 μl each). After two weeks, mice were sacrificed by cervical dislocation and LV myocytes were isolated as described previously in.^4^

### 2.3 Myocyte staining and immunofluorescence

Selective labelling and visualization of LV myocytes sarcolemmal and t-tubular membrane was achieved by using the cell-impermeant Alexa Fluor 594 wheat germ agglutinin (WGA, Molecular Probes) and a Zeiss 510 MetaHead confocal microscope. LV myocytes isolated from transduced hearts were fixed in 2% PFA for 5 min and permeabilized in 0.2% Triton x-100 for 30 min. Myocytes were blocked subsequently in 5% BSA for 1 h and incubated overnight with mouse monoclonal nNOS antibody (Santa Cruz Biotechnology). Samples were then washed with PBS and incubated with a secondary antibody. Finally samples were mounted with the addition of 10 µl of SlowFade Gold Antifade reagent (Invitrogen).

### 2.4 LV myocyte contractility, [Ca2+]i transients and I_Ca_

Cell shortening and the maximum rate of re-lengthening were measured in LV myocytes field-stimulated at 3 Hz by using a video-edge detection system (IonOptix Corp). The [Ca^2+^]_i_ transient was measured in fura-2 loaded (5 μmol/L, Molecular Probes) myocytes field-stimulated at 3 Hz. The amplitude of the [Ca^2+^]_i_ transient was calculated as the difference between diastolic and peak Ca^2+ ^fluorescence. The rate of decay of the field-stimulated [Ca^2+^]_i_ transient was best fit by a double exponential (Clampfit, Axonpatch, Axon Instruments) and Tau1 was used for comparisons between groups. The L-type Ca^2+ ^current (*I*_Ca_) was recorded by whole-cell patch clamp, as described previously in.^1^ Briefly, LV myocytes were placed in an experimental bath set on the stage of an inverted microscope and perfused with Tyrode solution containing in mmol/L: 140 TeaCl, 5.4 CsCl, 1.2 MgCl_2_, 5 HEPES, 11 glucose, 1.4 CaCl_2_, pH 7.4 with CsOH. Patch-clamp pipettes, prepared from glass capillary tubes by means of a two-stage horizontal puller (P-97 Flaming/Brown micropipette puller, Sutter Instrument, Novato, CA, USA), had a resistance of ∼2.5 MΩ when filled with pipette solution (containing in mmol/L: Cs-Aspartate 120, TEACl 10, MgATP 5, MgCl_2_ 2, CaCl_2_ 5, EGTA 11, HEPES 10 (pH 7.2 with CsOH). Recordings were performed by using a patch amplifier (Axopatch 200B, Molecular Devices, Sunnyvale, CA, USA) in whole-cell configuration. Signals were digitized via a DAC/ADC interface (Digidata 1200B, Molecular Devices) and acquired using the pClamp software. Whole cell patching was carried out only if the series resistance (Rs) was <15 mΩ. No Rs correction was applied.

*I*_Ca_ amplitude was measured by applying a voltage protocol for steady-state activation from –35 to +50 mV for 200 ms from a holding potential of –40 mV, to inactivate the sodium current. Peak *I*_Ca_ was measured as the difference between the peak inward current at the beginning of the depolarizing step and the steady-state current at the end of the step, normalized with respect to cell membrane capacitance (measured by applying a ±10 mV pulse of 18 ms, starting from a holding potential of –70 mV) and expressed as current density in pA/pF. Steady state inactivation was recorded during a test step at 0 mV following 500 ms conditioning steps at potentials between −75 and 5 mV. β-adrenergic receptor stimulation was achieved by adding isoproterenol (10–100 nmol/L) to the perfusate and nNOS inhibition by adding S-methyl-L-thiocitrulline (SMTC, 100 nmol/L) both to the perfusing and pipette solution (when applicable). All experiments were conducted at 35°C.

### 2.5 Biopterins quantification and NOS activity

Chromatographic analysis of BH4, 7,8-dihydrobiopterin (BH2) and biopterin (B) content in homogenized tissue using HPLC was carried out as described previously in.^24^ Biopterins were separated using a Carbon-18 column (Hichrom) and quantified using sequential electrochemical (Coulochem III, ESA Inc.) and fluorescence (Jasco) detection. Standards of BH4, BH2, and B were injected to calculate the final concentration and results were normalized to the protein content.

NOS activity was measured as the L-arginine to citrulline conversion by HPLC, as described previously in.^4^

### 2.6 Superoxide production

Myocardial superoxide production (Tiron-inhibitable fraction) was evaluated by using lucigenin (5 μmol/L)-enhanced chemiluminescence in LV homogenates, as described and validated previously in.^25^^,^^26^

### 2.7 Immunoblotting and immunoprecipitation assays

Immunoblotting in LV homogenates was performed using primary antibodies raised against NOS enzymes, Cav-3, the Ca^2+^ ATPase (SERCA2A), the Na^+^-Ca^2+ ^exchanger (NCX1), GAPDH (Santa Cruz Biotechnology), RyR2 (Thermo Fisher Scientific) and total and Ser^16^- phosphorylated phospholamban (PLB, Badrilla). For immunoprecipitation assays, LV were homogenized in IP buffer (1%Triton X-100, 150mM NaCl, 10mM Tris-HCl pH 7.4, 1mM EDTA, 1 mM EGTA and a protease inhibitor cocktail from Roche Diagnostics) and 350 µg of protein were precleared using 25 µl of Protein A/G–conjugated agarose. Samples were incubated overnight at 4 °C with primary antibodies at a final concentration of 10 µg/ml. Protein A/G –conjugated agarose was used to precipitate the primary antibodies for an additional 4 h, washed three times in IP buffer and then boiled in 60 µl of loading buffer. Samples were then subjected to SDS polyacrylamide gel electrophoresis and immunoblotting. The same membrane was first blotted for iNOS, then stripped and re-probed for eNOS. Similarly, we first blotted for phospho Ser^16^-PLB and then for total PLB, after stripping the membrane.

### 2.8 RyR2 receptor S-nitrosation

The biotin switch method was performed on LV homogenates, according to Jaffrey *et al*.,^27^ using the S-nitrosation Protein Detection Assay Kit (Cayman Chemical, USA). All steps were carried out according to the manufacturer’s protocol. For direct detection of biotinylated RyR2, 40 µg of each sample was subjected to SDS-PAGE using NuPage 4-12% Bis-Tris gels (Invitrogen), followed by immunoblotting with RyR2 antibody (Thermo Fisher Scientific; 1:500 in 2.5% milk). After stripping, membranes were re-probed with horseradish peroxidase-conjugated biotin antibody (Cell Signaling Technologies, 1:1000 in 0.1% BSA).

### 2.9 Statistics

Data are expressed as mean ± S.E.M. or median and 95% CIs. Results were analysed using ANOVA with Bonferroni correction, the Student’s *t*-test or non-parametric tests, as appropriate. A *P-*value < 0.05 was considered significant.

## 3. Results

### 3.1 The nNOS.eGFP protein is targeted to the sarcolemmal membrane

**Figure 1:**
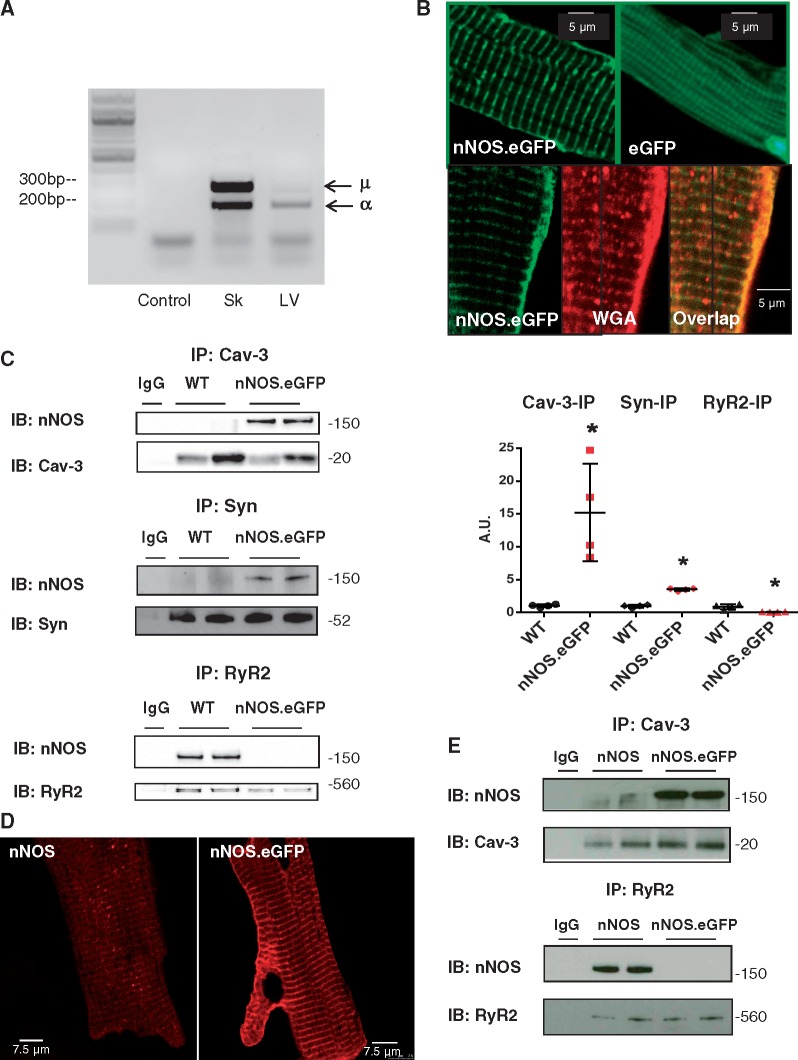
Myocardial nNOS expression and subcellular localization. *(A)* mRNA expression of α and μ nNOS isoforms in murine skeletal muscle and LV. Amplification of LV cDNA without primers was used as a negative control (*n* = 3 independent experiments per group). *(B)* Fluorescence from myocytes transduced with *nNOS*.eGFP (green, *top left panel*) show a striated and sarcolemmal pattern whereas fluorescence from myocytes transduced with eGFP *(top right panel*) is diffuse in the cytosol. Fluorescence from myocytes transduced with *nNOS*.eGFP (green, *bottom left panel*) and from the Alexa Fluor 594 conjugate of WGA (red, *bottom middle panel*) and their overlapped image (*bottom right panel*) confirms localization of nNOS to the sarcolemmal/t-tubular membrane (*n* = 8 independent experiments per group). *(C) Left panels*: Immunoprecipitation of syntrophin, Cav-3, or RyR2 followed by immunoblotting with an anti-nNOS antibody indicates predominant localization of nNOS.eGFP to the sarcolemmal membrane. Immunoprecipitation with anti-immunoglobulin G antibodies was used as negative control. *Right panel*: Scatterplot and average results; for each sample, the intensity of the nNOS band (*top membrane*) was normalized to that of the protein used in the immunoprecipitation (*bottom membrane*). The results are presented as fold difference from WT (* *P* < 0.05, *n* = 4 hearts per group; Mann-Whitney test). *(D)* Immunofluorescence showing nNOS localization in nNOS^−/−^ myocytes transduced with AdV.*nNOS (left panel)* or AdV.*nNOS*.eGFP *(right panel)*; 3 independent experiments per group. *(E)* Immunoprecipitation using a Cav-3 or RyR2 antibody in LV samples transduced using AdV.*nNOS* or AdV.*nNOS*.eGFP (2 independent experiments per group).

The α and μ isoforms of nNOS are the most abundant splice variants in neuronal tissue and striated muscle, respectively.^22^^,^^23^ Both isoforms share a similar sequence and structure (with μ being slightly longer than α due to a 34 amino acid insertion between the calmodulin- and flavin- binding domains^23^) and contain a N-terminal PDZ domain that allows them to localize to specific membrane regions.^28^^,^^29^ *Figure 1A* shows that both μ and α nNOS isoforms are expressed in the LV of WT mice. We carried out *in vivo* gene transfer using a replication-deficient AdV encoding for a human α-nNOS.eGFP fusion protein or eGFP only in the LV myocardium of nNOS^−^^/^^−^ mice. To assess the subcellular localization of nNOS.eGFP after AdV delivery, we stained the sarcolemmal and t-tubular membrane of transduced nNOS^−^^/^^−^ cardiomyocytes using the cell-impermeant Alexa Fluor 594 WGA. As shown in *Figure 1B*, the nNOS.eGFP fluorescence (green) overlapped with that of WGA (red), indicating a predominant sarcolemmal localization. In contrast, the eGFP fluorescence was diffused throughout the cell (*Figure 1B*). To establish the subcellular localization of nNOS.eGFP in more detail, we performed co-immunoprecipitation and found that whereas sarcolemmal-bound proteins, such as syntrophin and Cav-3, co-immunoprecipitated with nNOS.eGFP, RyR2 did not, confirming the preferential localization of nNOS.GFP to the sarcolemmal membrane (*Figure 1C*), as previously observed in remodelled and failing hearts.^18–20^ This result was confirmed by repeating the experiments in reverse order, i.e. by immunoprecipitating first with an anti-nNOS antibody and then blotting for Cav-3 and RyR2 (Supplementary material online, *Figure S1A*). It should be noted that a small pool of nNOS is also localized to the sarcolemmal membrane in WT myocytes (Supplementary material online, *Figure S1B*); however, this is not obvious in *Figure 1C* due to the more robust signal from the nNOS.eGFP transduced nNOS^−^^/^^−^ hearts.

Fusion of autofluorescent tags, such eGFP, with cellular proteins facilitates their detection and provides information about their intracellular localization. However, the tag may mask targeting signals contained within the expressed protein restricting the range of its subcellular localization.^30^ To evaluate whether the C-terminus eGFP tag was responsible for the preferential sarcolemmal localization of nNOS in nNOS^−^^/^^−^ mice, we transduced nNOS^−^^/^^−^ hearts with an AdV encoding for α-nNOS without the tag (AdV.*nNOS*). As shown in *Figure 1D*, immunohistochemistry suggests that intracellular localization of nNOS and nNOS.eGFP may differ. Indeed, co-immunoprecipitation in AdV.*nNOS* transduced hearts confirmed a predominant localization of nNOS to the SR membrane in association with RyR2 (*Figure 1E*), as described in WT mice.^13^ Taken together, these findings indicate that the presence of the C-terminus eGFP tag selectively targets nNOS to the sarcolemma membrane, thereby providing a model in which to study the impact of nNOS subcellular localization on myocardial EC coupling.

### 3.2 Adenoviral gene transfer of nNOS.eGFP in the LV myocardium of nNOS^−^^/^^−^ mice increases NOS activity and decreases superoxide production

**Figure 2:**
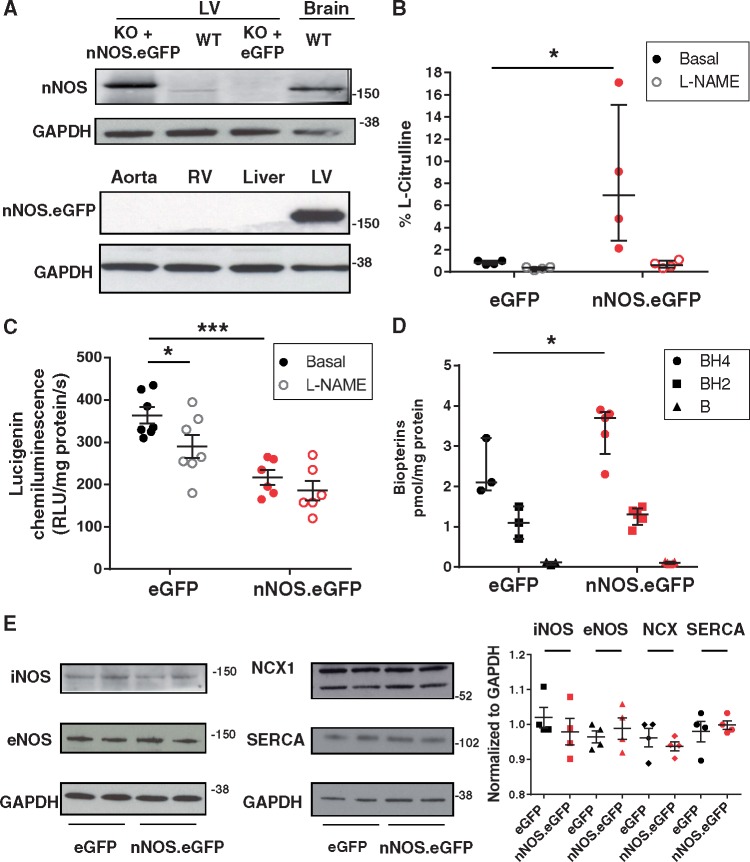
Effect of *nNOS*.eGFP on the myocardial nitroso-redox balance of nNOS^−/−^ mice. *(A)* nNOS protein expression in LV homogenates from nNOS^−/−^ mice transduced with either AdV.*nNOS*.eGFP or AdV.eGFP. Brain homogenate was used as a positive control. The nNOS.eGFP fusion protein is only detected in the LV myocardium (*n* = 4 independent experiments per group). *(B)* NOS activity is significantly increased in nNOS^−/−^ hearts injected with AdV.*nNOS*.eGFP vs. AdV.eGFP (* *P* < 0.05, *n* = 4 and 5 hearts; Kruskal-Wallis) and significantly inhibited by L-NAME (1 mmol/L). *(C)* Myocardial superoxide production is significantly lower in *nNOS*.eGFP transduced nNOS^−/−^ mice (*** *P* < 0.001 vs. eGFP, *n* = 6 and 7 hearts; two-way ANOVA) where is no longer significantly decreased by NOS inhibition with L-NAME (1 mmol/L, * *P* < 0.05 vs. basal). *(D)* The LV BH4 content is significantly higher in *nNOS*.eGFP transduced hearts (* *P* < 0.05 vs. eGFP, *n* = 3 and 5 hearts, Mann-Whitney test) whereas the BH4 oxidized products (BH2 and B) are unchanged. *(E)* Western blotting showed no significant differences in iNOS, eNOS, NCX1 and SERCA2A between AdV.eGFP and AdV.*nNOS*.eGFP injected LV (*P* = 0.49, 0.66, 0.49, and 0.99, respectively, *n* = 4 hearts per group; Mann-Whitney test). Molecular weight in Kilo Daltons is shown on the right side of the blots.

*In vivo* AdV gene delivery of *nNOS*.eGFP or eGFP alone in nNOS^−^^/^^−^ mice yielded a transduction rate of *ca.* 30% (assessed by quantifying eGFP fluorescence in LV cryosections). Gene transfer was confined to the LV myocardium as indicated by failure to detect nNOS protein in other organs (*Figure 2A*). NOS activity in LV homogenates from *nNOS*.eGFP transduced nNOS^−^^/^^−^ mice was significantly higher than that of eGFP-transduced animals (*Figure 2B*).

We have previously reported that eNOS activity is uncoupled in the myocardium of nNOS^−/−^ mice secondary to an increase in enzyme S-glutathionylation.^9^ Consistent with these findings, NOS inhibition was associated with a significant reduction in superoxide production in LV lysates from nNOS^−/−^ mice transduced with eGFP (*Figure 2C*). In contrast, myocardial *nNOS*.eGFP overexpression was associated with a significant reduction in basal LV superoxide production (*Figure 2C*) and NOS inhibition with L-NAME (1 mmol/L) did not have a significant effect on LV superoxide measurements in *nNOS*.eGFP transduced mice (*Figure 2C*). The NOS co-factor tetrahydrobiopterin (BH4) was also significantly increased in the LV myocardium of *nNOS*.eGFP transduced mice whereas oxidized biopterins remained unchanged (*Figure 2D*).

Protein expression of the endothelial or inducible NOS isoform (eNOS and iNOS, respectively) did not differ between *nNOS*.eGFP and eGFP-transduced nNOS^−/−^ hearts; similarly, the abundance of other proteins involved in E–C coupling (e.g. SERCA2A and NCX) did not differ between groups (*Figure 2E*).

Together these findings indicate that neither the eGFP tag nor the AdV-mediated overexpression affected the ability of nNOS to synthesize NO. AdV-mediated sarcolemmal expression of nNOS.eGFP in the myocardium of nNOS^−/−^ mice resulted in an increase in coupled NOS activity and BH4 availability and a reduction in superoxide release, in the absence of changes in eNOS and iNOS protein level.

### 3.3 Sarcolemmal expression of nNOS in LV myocytes decreases basal and β-adrenergic contraction and I_Ca_ density

**Figure 3:**
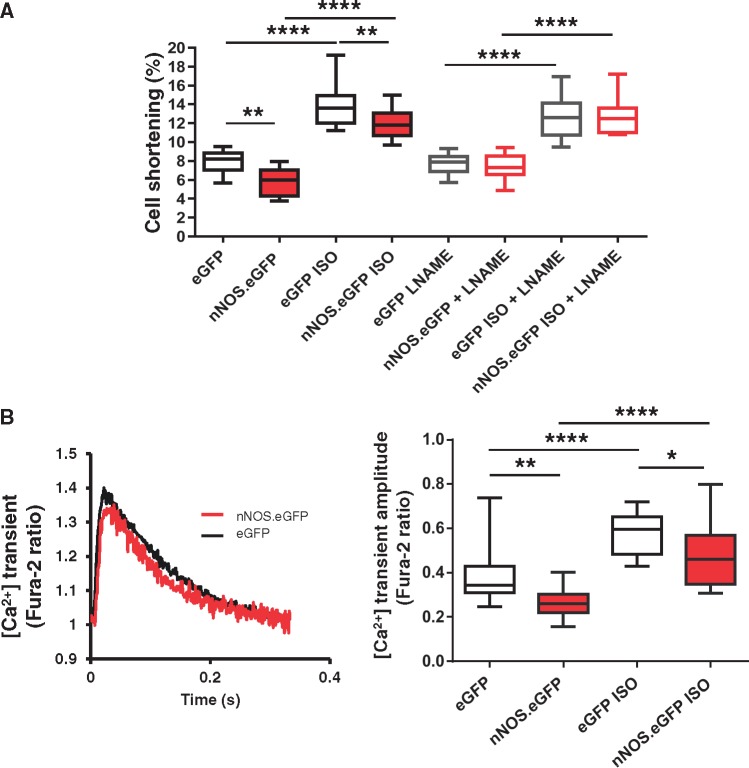
nNOS.eGFP reduces contractility and [Ca^2+^]_i_ transients in LV myocytes from nNOS^−/−^ mice. *(A)* Isoproterenol (ISO, 10 nmol/L) increased cell shortening in both *nNOS*.eGFP and eGFP expressing myocytes (****P < 0.0001 vs. Basal, *n* = 12–21 cells, four hearts per group; two-way ANOVA). Both basal and ISO-stimulated cell shortening were lower in AdV.*nNOS*.eGFP transduced myocytes compared with AdV.eGFP controls (**P < 0.01, two-way ANOVA). These differences were lost after incubation with L-NAME (1 mmol/L, *n* = 10–14 cells, three hearts per group). *(B)* Representative [Ca^2+^]_i_ transients obtained in fura-2 loaded, field-stimulated (3 Hz, 35 °C) LV myocytes from *nNOS*.eGFP- or eGFP-transduced nNOS^−/−^ hearts. ISO (10 nmol/L) increased the amplitude of the [Ca^2+^]_i_ transients in both groups (****P < 0.0001 vs. Basal, *n* = 12–23 cells from four hearts; two-way ANOVA); however, the amplitude of the [Ca^2+^]_i_ transients was significantly lower in nNOS^−/−^ myocytes transduced with AdV.*nNOS*.eGFP both under basal conditions and in the presence of ISO (*P < 0.05, **P < 0.01 vs. eGFP; two-way ANOVA).

We have previously shown that nNOS inhibition or gene deletion is associated with increased basal and β-adrenergic cell shortening and prolonged relaxation.^1^^,^^5^^,^^31^ Sarcolemmal expression of the nNOS.eGFP protein significantly decreased cell shortening under basal conditions and in the presence of β-adrenergic stimulation with 10 nmol/L isoproterenol (*Figure 3A*). We also collected data from LV myocytes from the same isolation that had not been transduced during the 2-week incubation period (non-infected controls). Cell shortening in these myocytes was not different from that measured in cells transduced by AdV.eGFP (7.5 ± 0.3% vs. 8.0 ± 0.4% in eGFP-transduced nNOS^−/−^ myocytes; *n* = 12–30, P = NS). Reduced cell shortening in *nNOS*.eGFP transduced LV myocytes was associated with a lower [Ca^2+^]_i_ transient amplitude both under basal conditions and in the presence of isoproterenol (*Figure 3B*).

**Figure 4:**
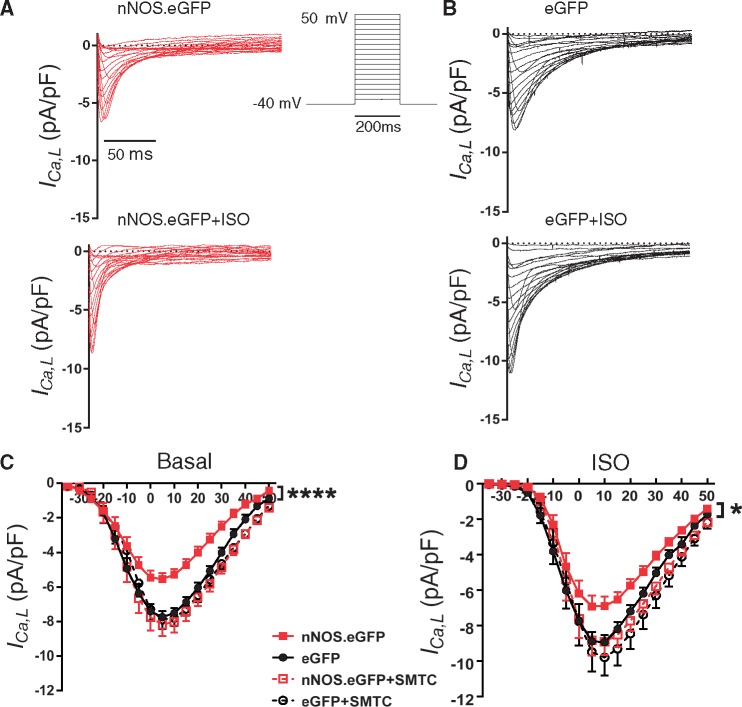
nNOS.eGFP reduces *I*_Ca_ in LV myocytes from nNOS^−/−^ mice. Voltage protocol and examples of L-type Ca^2+^ current (*I*_Ca_) recordings under basal condition and in the presence of ISO in nNOS^−/−^ LV myocytes transduced with *nNOS*.eGFP *(A)* or eGFP *(B)*. Capacitive currents have been removed for clarity. *(C)* The current-voltage relationship of *I*_Ca_ showed a significantly larger current density in nNOS^−/−^ LV myocytes transduced with AdV.eGFP *vs. nNOS*.eGFP; ****P < 0.0001, *n* = 31 and 23 cells from 12 hearts; two-way ANOVA). nNOS inhibition with SMTC (100 nmol/L) resulted in a larger *I*_Ca_ in nNOS.eGFP expressing myocytes only (empty red squares) and abolished the difference between groups (*n* = 17 and 18 cells from 11 hearts). *(D)* In the presence of isoproterenol, *I*_Ca_ density was larger in nNOS^−/−^ LV myocytes transduced with AdV.eGFP vs. AdV.*nNOS*.eGFP (*P < 0.05, *n* = 8 cells from eight hearts per group; two-way ANOVA). These differences were abolished after nNOS inhibition with SMTC.

To assess whether an increase in myocardial NO production was directly implicated in these findings, we compared cell shortening after acute NOS inhibition with L-NAME (1 mmol/L). L-NAME did not affect contraction in eGFP-transduced or non-transduced myocytes from nNOS^−^^/^^−^ hearts but restored basal and β-adrenergic contraction in nNOS.eGFP expressing myocytes (*Figure 3A*). Sarcolemmal nNOS.eGFP expression also resulted in a lower *I*_Ca_ density both under basal conditions (*Figure 4**A and C*) and in the presence of isoproterenol (100 nmol/L; *Figure 4**B and D*). nNOS inhibition with SMTC (100 nmol/L) increased *I*_Ca_ in *nNOS*.eGFP transduced myocytes only, and abolished the differences between groups under basal conditions and in the presence of isoproterenol (*Figure 4*). Steady-state activation curves showed that the voltage at which *I*_Ca_ was half-maximally activated did not differ between groups (Supplementary material online. *Fig 1C*). In contrast, the voltage of half–maximal inactivation of *I*_Ca_ in nNOS.eGFP transduced myocytes was shifted to the left (*V*_1/2_: −36 ± 1 mV vs. −32 ± 1 mV in eGFP; *n* = 11 and 15 cells, *P* < 0.05; Supplementary material online, *Figure S2A*) under basal conditions, further contributing to the reduction in Ca^2+ ^influx in this group. This difference was abolished after incubation with SMTC or in the presence of isoproterenol (Supplementary material online, *Figure S2B*).

**Figure 5:**
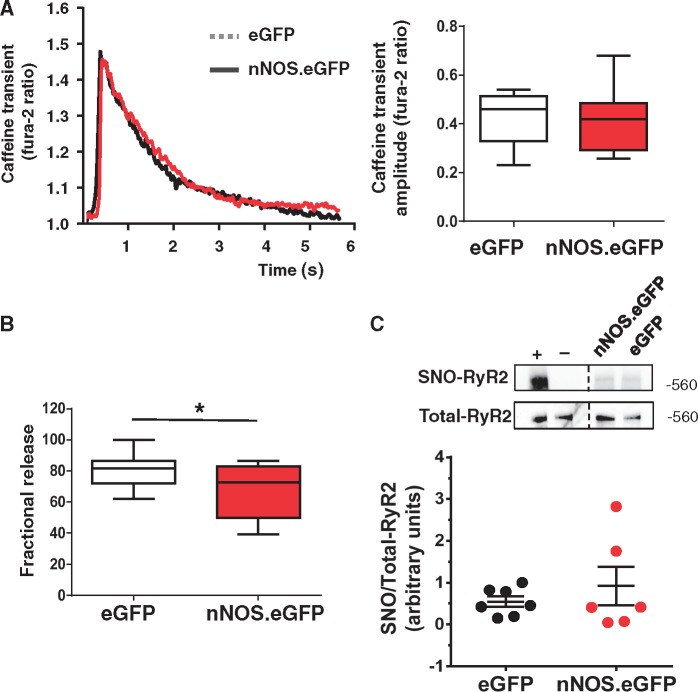
Impact of sarcolemmal nNOS expression on SR Ca^2+ ^load and fractional release, and ryanodine receptor *S*-nitrosation. *(A) Left panel*: Representative traces of the caffeine-induced [Ca^2+^]_i_ transients of nNOS^−/−^ LV myocytes expressing nNOS.eGFP or eGFP. *Right Panel*: Average data showed no significant difference in the amplitude of the caffeine [Ca^2+^]_i_ transients between groups, (*P* = NS, *n* = 10–14 cells from four hearts per group; Student’s *t*-test). *(B)* Fractional Ca^2+ ^release was significantly lower in LV myocytes expressing nNOS.eGFP (*P < 0.05, *n* = 10–14 cells from four hearts per group; Student’s *t*-test). *(C)* Ryanodine receptor *S*-nitrosation (SNO-RyR2) did not differ between AdV.eGFP and AdV.*nNOS*.eGFP transduced hearts (*P* = NS, *n* = 6 per group; Mann-Whitney test). Positive control (+): homogenates treated with S-nitrosoglutathione (500 µM) prior to performing the biotin switch. Negative control (−): the biotin tag was not added during the labelling reaction. Images were cropped from the same membrane where indicated by the dashed line. Representative Western blots using an anti-biotin antibody (*top*) or RyR2 antibody (*bottom*). The bar chart shows the average ratio of biotin to RyR2.

SR Ca^2+ ^content did not differ between groups (*Figure 5A*) but the reduction in *I*_Ca_ in LV myocyte expressing nNOS.eGFP was associated with a smaller fractional release of Ca^2+ ^from the SR (*Figure 5B*). The presence of nNOS.eGFP in the sarcolemmal membrane did not alter S-nitrosation of the RyR2 significantly (*Figure 5C*), indicating that direct effects of nNOS-derived NO on the RyR2 require co-localization of the respective proteins. Collectively, these data indicate that sarcolemmal nNOS expression reduces cell shortening and the amplitude of the Ca^2^^+^ transient by decreasing Ca^2+ ^influx *via* the L-type Ca^2+ ^channel.

### 3.4 Sarcolemmal expression of nNOS does not affect relaxation or the rate of decay of [Ca^2+^]_i_ transients

**Figure 6:**
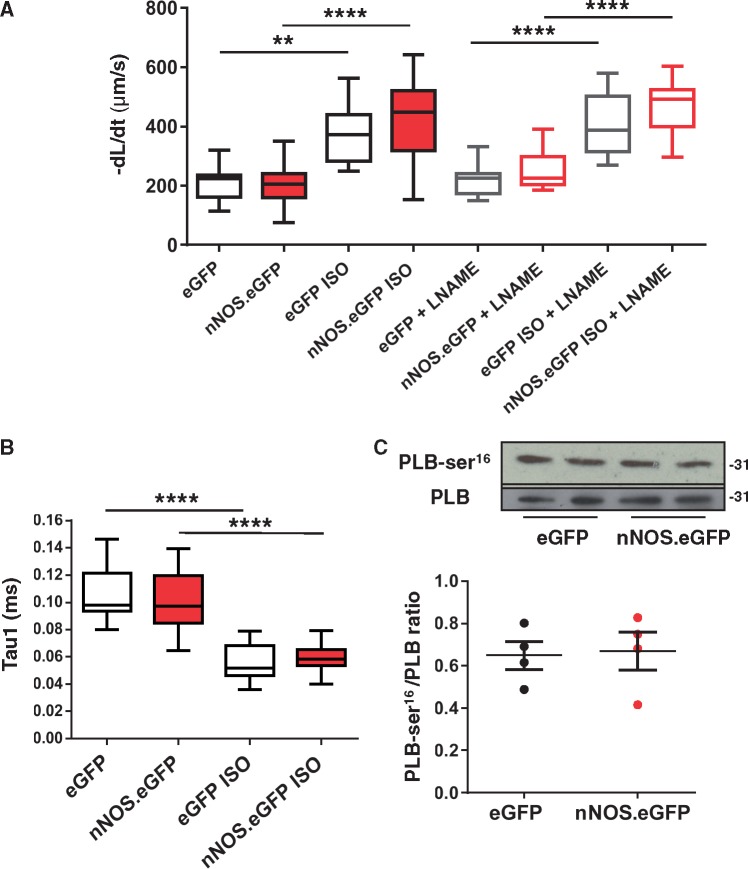
Myocyte relaxation is unaltered by nNOS.eGFP expression. *(A)* Returning velocity (dl/dt, μm/s) is faster in the presence of ISO in both nNOS.eGFP and eGFP overexpressing LV myocytes in the presence or absence of L-NAME (**P < 0.01; ****P < 0.0001 vs. basal, *n* = 11–19 cells from four hearts; two-way ANOVA) but is unaltered by nNOS.eGFP expression (*P* = NS vs. eGFP; two-way ANOVA) or by L-NAME (*P* = NS, *n* = 8–12 cells from three hearts per group; two-way ANOVA). *(B)* Similarly, the rate of decay of the [Ca^2+^]_i_ transients (Tau1) was significantly faster in the presence of ISO in both groups (*n* = 10–18 cells from four hearts per group; two-ANOVA, **** *P* < 0.0001 vs. basal) but did not differ between *nNOS*.eGFP and eGFP overexpressing LV myocytes (*P* = NS, *n* = 10–18 cells from four hearts per group; two-way ANOVA). *(C)* Western blotting showed no significant differences in total and serine 16-phosphorylated PLB between eGFP and nNOS.eGFP expressing hearts (*P* = NS, *n* = 4 per group, Mann-Whitney test). Molecular weight (shown on the right side of the immunoblots) is expressed in Kilo Daltons.

Stimulation of nNOS activity in the SR hastens relaxation by increasing the PLB phosphorylated fraction.^4^ In contrast, sarcolemmal expression of nNOS.eGFP was not associated with a significant change in the rate of myocytes relaxation both under basal conditions and in the presence of β-adrenergic stimulation with isoproterenol, before or after pre-incubation with L-NAME (*Figure 6A*). As expected, β-adrenergic stimulation with isoproterenol significantly shortened the time constant of decay of [Ca^2+^]_i_ transient (Tau1) in both groups (*Figure 5B*); however, Tau1 did not differ between eGFP.*nNOS* and eGFP transduced myocytes both under basal conditions and in the presence of isoproterenol (*Figure 6B*). In keeping with these findings, total PLB and its Ser^16^ phosphorylated fraction did not differ between nNOS^−/−^ myocytes expressing sarcolemmal nNOS.eGFP and those expressing eGFP only (*Figure 6C*).

## 4. Discussion

It has been suggested that preferential sarcolemmal localization of nNOS in failing hearts may play an important role in the downstream effects of NO on cardiac function.^18–21^ Here we show that injection of AdV.*nNOS*.eGFP into the LV myocardium of nNOS^−/−^ mice results in sarcolemmal nNOS expression and in an increase in NOS activity and BH4 availability, in the absence of changes in oxidized biopterins. LV superoxide production is lower in *nNOS*.eGFP transduced mice and unaffected by L-NAME, indicating that nNOS activity remains coupled under these conditions. Importantly, sarcolemmal nNOS expression is associated with a reduction in *I*_Ca_ current, fractional release of Ca^2+ ^from the SR, and in the amplitude of the [Ca^2+^]_i_ transient leading to lower basal and β-adrenergic cell shortening, compared with myocytes transduced with eGFP only. In contrast, S-nitrosation of the RyR2, PLB phosphorylation, and the rate of myocyte relaxation, [Ca^2+^]_i_ decay remained unaltered in *nNOS*.eGFP transduced myocytes. These findings indicate that diffusibility of NO in the LV myocardium is limited and subcellular localization of nNOS determines the downstream targets of NO and its effect on E–C coupling. They also suggest that translocation of nNOS to the sarcolemmal membrane, as described in human and experimental heart failure,^18^^,^^19^^,^^21^ may protect the myocardium against the effect of neurohumoral activation at the expense of impairing LV diastolic function.

### 4.1 Myocardial nNOS expression and subcellular localization in health and disease

Among the NOS isoforms, nNOS is the only one to possess a PDZ domain at the N-terminus through which it interacts with a variety of proteins in specific subcellular domains. In the healthy myocardium, nNOS is mainly (but not exclusive) localized to the SR membrane where it has been found to co-localize with the RyR2 and the Ca^2+ ^ATPase, SERCA2A.^13^^,^^14^^,^^32^ In a rat model of post-infarction LV remodelling and in failing human hearts nNOS expression and activity have been reported to increase.^18–20^ Under these circumstances, nNOS appears to translocate to the sarcolemma in a process that implicates Cav-3 and the heat shock protein 90.^18–20^ An increase in sarcolemmal nNOS has also been observed in mice following ischemia/reperfusion in the presence of β-adrenergic stimulation.^21^ The functional correlates of this phenomenon have been difficult to unravel from the changes brought about by the underlying disease state. Bendall *et al.*^20^ reported a role for nNOS in the depressed β-adrenergic reserve of post-infarction rats whereas Sun *et al.*^21^ showed an increase in S-nitrosation of the L-type Ca^2^^+^ channel following ischemia/reperfusion, resulting in a reduction in Ca^2^^+^ entry and SR Ca^2^^+^ loading that was more accentuated in female mice and dependent on both nNOS and eNOS activity.

We expressed *nNOS*.eGFP in the myocardium of nNOS^−/−^ mice using *in vivo* AdV gene transfer and exploited the ability of the eGFP tag to restrict the subcellular localization of fusion proteins^30^ (usually by steric hindrance or interruption of critical C and -terminal localization/retention sequences^33^^,^^34^) to investigate the role of nNOS-derived NO at the myocyte sarcolemmal membrane. AdV-gene transfer of eGFP only resulted in diffuse fluorescence and no difference in myocyte cell shortening (compared with non-transduced LV myocytes from the same isolation). In contrast, *nNOS*.eGFP transduced nNOS^−^^/^^−^ LV myocytes showed a reduction in basal and β-adrenergic contraction.

Previous reports of myocardial transgenic overexpression of nNOS did not show consistent effects on myocardial contractility. Burkard *et al.*^32^ showed that conditional myocardial-restricted overexpression of α-nNOS resulted in a six-fold increase in nNOS protein, which co-localized with both SERCA2A and the L-type Ca^2+ ^channel. A 30% increase in total NOS activity was associated with a reduction in both basal and dobutamine-stimulated LV function that was mimicked in isolated LV myocytes and associated with a reduction in *I*_Ca_. However, despite parallel overexpression of nNOS in the proximity of SERCA2A, they observed slower relaxation and decay of the [Ca^2+^]_i_ transient and reduced PLB phosphorylation in induced mice. Even more puzzling, in another mouse model of conditional nNOS overexpression, resulting in an increase in both Cav-3 and RyR2-associated nNOS, cell shortening and [Ca^2+^]_i_ transient amplitude increased whereas the decay of the [Ca^2+^]_i_ transient was significantly faster. The reasons underlying this discrepancy are not clear; however, NOS overexpression can result in a relative reduction in the availability of the enzyme’s co-factor BH4, leading to NOS uncoupling, increased superoxide production, and unpredictable functional consequences.^35^^,^^36^ To exclude this possibility, we measured BH4 and its oxidized products and the L-NAME-inhibitable superoxide production (a hallmark of NOS uncoupling^9^) in LV homogenates from nNOS.eGFP and eGFP transduced nNOS^−/−^ mice. Our results show that sarcolemmal AdV nNOS.eGFP expression is associated with a modest increase in BH4, a reduction in superoxide production, and in an increase in NOS activity. We have previously reported that nNOS gene deletion is associated with myocardial eNOS uncoupling.^9^ Here we show that the L-NAME-inhibitable superoxide production observed in the LV of eGFP transduced nNOS^−/−^ mice is no longer detectable in *nNOS*.eGFP transduced mice. The latter showed a reduction in total LV superoxide production that was unaffected by L-NAME, suggesting that the mechanism underlying the antioxidant effect of sarcolemmal nNOS expression in the LV of nNOS^−^^/^^−^ mice is not acutely dependent on NO synthesis but may depend on the ability of nNOS to link components of signal transduction pathways by using its N-terminus PDZ domain.^37^ Indeed nNOS has been found to co-localize with xanthine oxidase and inhibit production of superoxide from this oxidase system.^7^^,^^8^

### 4.2 nNOS regulation of myocardial EC coupling

We and others have previously reported that nNOS gene deletion or pharmacological inhibition results in greater cell shortening, *I*_Ca_ and [Ca^2+^]_i_ transient amplitude, and a slower rate of relaxation and SR Ca^2+ ^reuptake, secondary to a reduction in PLB phosphorylation^1^^,^^2^^,^^5^^,^^6^ whereas increasing nNOS activity (achieved by increasing myocardial BH4 availability) reduces *I*_Ca_ density and hastens relaxation by increasing PLB phosphorylation.^4^ In this study sarcolemmal-selective expression of nNOS.eGFP in the myocardium of nNOS^−/−^ mice recapitulated the effect of nNOS-derived NO on basal and β-adrenergic contraction and *I*_Ca_ in the absence of changes in the protein content of iNOS and eNOS or of calcium handling proteins, such as the NCX and SERCA2A. In contrast, sarcolemmal nNOS.eGFP did not alter PLB phosphorylation, the rate of Ca^2+ ^reuptake or relaxation in nNOS^−/−^ myocytes. nNOS has also been reported to regulate RyR2 activity via S-nitrosation.^3^ RyR2A S-nitrosation is reduced in nNOS^−/−^ mice and in the presence of heart failure, where nNOS is overexpressed but predominantly localized to the sarcolemmal membrane.^38^^,^^39^ Our results suggest that the reduction in cell shortening observed in myocytes expressing sarcolemmal *nNOS*.eGFP is unlikely to be caused by changes in RyR2 S-nitrosation, confirming that subcellular localization of nNOS is instrumental to the interaction of NO with specific targets.

Collectively, our findings highlight the importance of nNOS subcellular localization on its downstream effects in the myocardium. Indeed, myoglobin is expected to act as a potent ‘sink’ for NO in cardiomyocytes, limiting its diffusion between different intracellular microdomains and keeping the actions of NO circumscribed to a very small locale. The fact that nNOS-derived NO signalling is mostly mediated by S-nitrosation of its targets rather than by cGMP signalling,^3^^,^^21^^,^^40^ may further support a localized mode of action. Once located to the sarcolemmal membrane NO may nitrosylate the L-type Ca^2+ ^channels and be more likely to exert paracrine actions by diffusing outside the myocyte, as described in the skeletal muscle^41^, than to reach other intracellular targets.^42^ The importance of nNOS localization has also been highlighted in a recent study of mechano-chemotransduction in LV myocytes where it was again shown that the effective range of intracellular NO signalling is very limited; in particular, they reported that whereas nNOS-derived NO is able to regulated RyR2 function due to the proteins’ close proximity, sarcolemmal eNOS-derived NO had no effect.^43^

In summary, our findings demonstrated the importance of nNOS subcellular localization in determining NO signalling targets in the LV myocardium and suggest that preferential sarcolemmal localization of nNOS in failing hearts may protect the myocardium from excessive sympathetic stimulation at the expense of reducing PLB phosphorylation and impairing LV diastolic function.

## Supplementary material

Supplementary material is available at *Cardiovascular Research* online.

**Conflict of interest**: none declared.

## Funding

This work was supported by a Programme Grant of the British Heart Foundation (RG/11/15/29375).

## Supplementary Material

Supplementary Data
