# Supplementary material for: The subcellular localization of neuronal nitric oxide synthase determines the downstream effects of NO on myocardial function
Source: Cardiovasc Res. 2017 Feb 1;113(3):321–31. doi: 10.1093/cvr/cvx002 (PMC5408949; doi:10.1093/cvr/cvx002)
Supplement: Supplementary Data [file cvx002_Supp.pdf]

Supplementary Figure 1

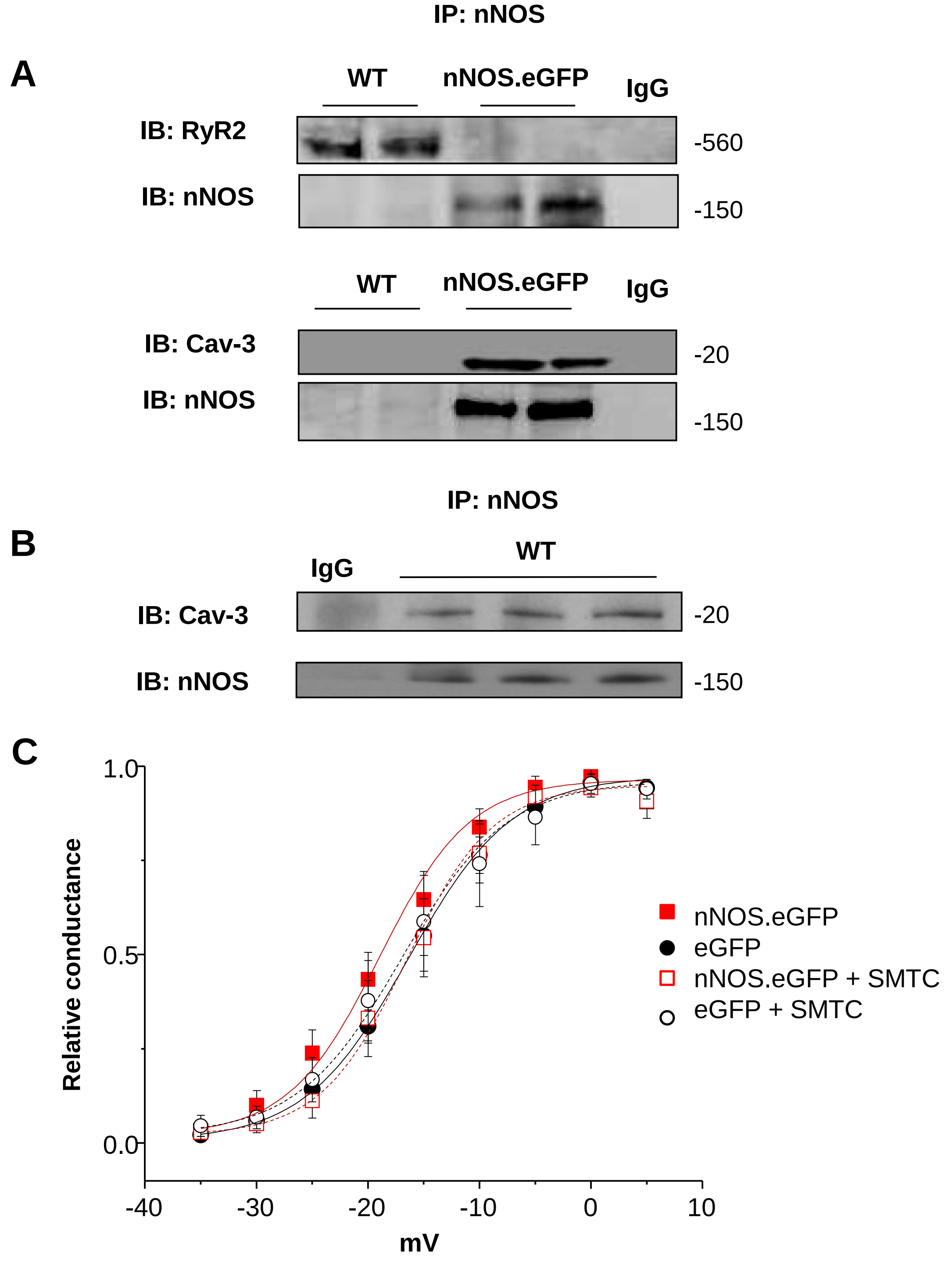

Supplementary Figure 1.
(**A**) Immunoprecipitations of RyR2 and caveolin-3 (Cav-3) followed by immunoblotting with an anti-nNOS antibody demonstrate predominant localization of native nNOS (WT, wild type) to the sarcoplasmic reticulum and of nNOS.eGFP to the sarcolemmal membrane (n=4 hearts). IgG: negative control.
(**B**) In WT LV homogenates, a small amount of caveolin-3 is immunoprecipitated with nNOS (n=3).
(**C**) Steady-state activation curves showed that the voltage at which I_{Ca} was half-maximally activated was not significantly different between groups (-15.6±0.6 mV in eGFP *vs.* -18.9±0.9 mV in nNOS.eGFP, n=14-18 cells from 4 hearts and -15.8±0.6 in eGFP *vs.* -16.1±0.6 mV in nNOS.eGFP in the presence of SMTC, n=6-10 cells from 4 hearts; ANOVA, P=NS).

Supplementary Figure 2

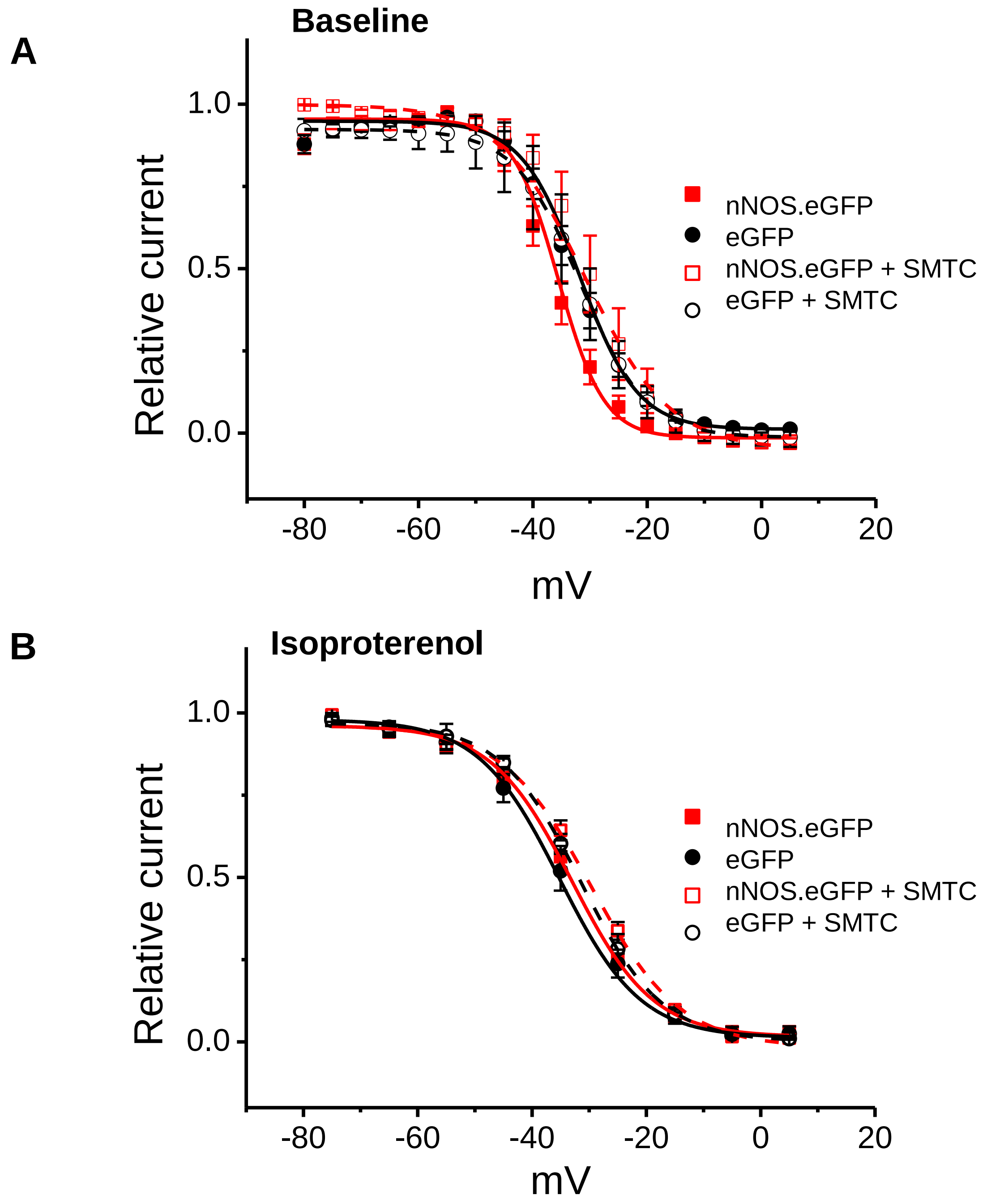

Supplementary Figure 2. (**A**) Under basal conditions, the voltage of half–maximal inactivation of I_{Ca} in nNOS.eGFP transduced myocytes was shifted to the left. This difference was abolished after nNOS inhibition with SMTC (P=NS, n=5 and 6 cells from 3 hearts). (**B**) In the presence of isoproterenol (100 nmol/L), inactivation curves did not differ between nNOS.eGFP (n=8 cells from 8 hearts) and eGFP expressing myocytes (n=7 cells from 7 hearts) in the presence and absence of SMTC. Under these conditions, the voltage at which I_{Ca} was half-maximally inactivated was -34.5±2.1mV in eGFP *vs*. -33.6±1.4 mV in nNOS.eGFP and -31.1±1.2 in eGFP *vs*. -29.9±1.2 mV in nNOS.eGFP in the presence of SMTC; ANOVA with Bonferroni's correction, P=NS).
